# The Emerging Roles of the Stress Epigenetic Reader LEDGF/p75 in Cancer Biology and Therapy Resistance: Mechanisms and Targeting Opportunities

**DOI:** 10.3390/cancers16233957

**Published:** 2024-11-26

**Authors:** Greisha L. Ortiz-Hernandez, Evelyn S. Sanchez-Hernandez, Pedro T. Ochoa, Carlos A. Casiano

**Affiliations:** 1 Center for Health Disparities and Molecular Medicine, Department of Basic Sciences, Loma Linda University School of Medicine, Loma Linda, CA 92350, USA; gortizhernandez@coh.org (G.L.O.-H.); esanchezhernandez@sbpdiscovery.org (E.S.S.-H.); pedroochoa@students.llu.edu (P.T.O.); 2 Department of Medicine, Division of Rheumatology, Loma Linda University School of Medicine, Loma Linda, CA 92350, USA; 3 Cancer Center, Loma Linda University Health, Loma Linda, CA 92350, USA

**Keywords:** LEDGF/p75, cancer, chemoresistance, DFS70, integrase binding domain, PSIP1, PWWP domain, RNA polymerase II, therapeutic targeting

## Abstract

The lens epithelium derived growth factor of 75 kD (LEDGF/p75) is a transcription co-activator and epigenetic reader that has emerged as a stress oncoprotein in multiple human cancers. Growing evidence indicates that it promotes tumor cell survival against certain therapeutic drugs. The amino (N)-terminal region of LEDGF/p75 contains a PWWP domain that reads methylated histone marks, critical for recognizing transcriptionally active chromatin sites. Its carboxyl (C)-terminus has an integrase binding domain (IBD) that serves as the binding site for the HIV-1 integrase and multiple oncogenic transcription factors. Acting as hubs for protein-protein interactions, both domains facilitate the tethering of oncogenic transcription factors and regulators to active chromatin to regulate mRNA splicing, promote DNA repair, and enhance the expression of stress and cancer-related genes that contribute to tumor cell aggressiveness and chemoresistance. This review summarizes our current knowledge of the emerging roles of LEDGF/p75 in cancer biology and therapy resistance and discusses its potential as a novel oncotherapeutic target in combinatorial treatments.

## Simple Summary

The nuclear protein lens epithelium derived growth factor of 75 kD (LEDGF/p75) has recently attracted considerable attention in the field of cancer research because of its high abundance in cancer cells and its role in promoting tumor aggressive properties. LEDGF/p75 is activated in cancer cells by agents that trigger cellular stress such as chemotherapeutic drugs. This protein has two major structural features, the PWWP domain and the IBD domain. These domains allow LEDGF/p75 to play important roles in cancer cells, including repairing damage to DNA, regulating the processing of messenger RNA, and binding to other nuclear proteins that are necessary to activate cancer-related genes involved in promoting cancer aggressiveness and resistance to therapy. This article discusses LEDGF/p75’s role in cancer and therapy resistance, and its potential as a therapeutic target for developing new combinatorial cancer treatments.

## 1. Introduction

The complexity of the RNA polymerase II (RNAPII) transcription machinery is critical for the regulation of gene expression [1,2,3]. This has been particularly documented in cancer cells, where interactions between RNAPII and transcription factors, co-activators, co-repressors, epigenetic readers, and chromatin remodelers orchestrate changes in gene expression during cancer initiation, progression, and response to therapy [4,5,6]. For example, the dysregulation, transcriptional activity, and chromatin interactions of the androgen receptor (AR) are critical for the activation of gene pathways associated with tumor progression in prostate cancer (PCa) [7]. Interestingly, the RNAPII transcriptional machinery of the cancer cells adapts to upregulate and recruit other transcription factors, such as glucocorticoid receptor (GR) and AR splice variants, to bypass blockade of AR transcriptional activity and continue driving disease progression and therapy resistance in patients treated with anti-AR therapies [7,8,9,10,11,12,13,14,15]. This transcriptional plasticity points to the ability of cancer cells to develop transcriptional addiction, which is necessary to maintain excessive proliferation and migration, metastasis, stress survival, and resistance to therapy [16].

The lens epithelium-derived growth factor protein of 75 kD (LEDGF/p75) is a transcriptional co-activator of RNAPII [17]. Although LEDGF/p75’s role as a growth factor for lens epithelial cells and other cells has been suggested [18,19,20], compelling evidence has revealed that this protein is a stress response epigenetic reader that tethers transcription factors, including several with relevance to cancer, to active chromatin sites [21,22]. LEDGF/p75 is induced by microenvironmental stressors that increase oxidative stress such as radiation, starvation, oncogenic viruses, oxidants, and cytotoxic drugs, and stimulates the upregulation of cellular protective genes [23,24,25,26,27,28,29,30,31,32,33,34,35,36,37,38]. Growing evidence indicates that its expression is upregulated in human cancers and promotes tumor cell aggressive properties such as increased proliferation, invasion, migration, clonogenicity, angiogenesis, DNA repair, and resistance to therapy-induced cell death [36,37,38,39,40,41,42,43,44,45,46,47,48,49,50,51].

LEDGF/p75 is also known as the dense fine speckled autoantigen of 70 kD (DFS70) and is the target of autoantibodies in subsets of healthy individuals and patients with diverse inflammatory conditions and cancer [52,53,54,55,56]. It also functions as a key cellular host factor used by the HIV-1 integrase (HIV-IN) to facilitate viral integration into active chromatin sites, leading to viral replication and assembly [57,58,59]. While the biological functions of LEDGF/p75 have been extensively reviewed in these disease contexts, there are currently no comprehensive reviews focusing on LEDGF/p75’s relevance to cancer. Here we discuss the role of LEDGF/p75 in cancer biology, emphasizing its emergence as a driver of cancer therapy resistance and potential as oncotherapeutic target.

## 2. LEDGF/p75 Discovery

LEDGF/p75 was discovered in 1998 by Roeder’s group and during the process of characterizing the general transcription co-activator PC4, two novel human transcriptional co-activators derived from alternative splicing, p75 and p52, were identified which interacted with the PC4 and RNAPII subunits [17]. Around the same time, Tan’s group reported that the sequence of transcription co-activator p75 corresponded to the sequence of their recently identified DFS70 autoantigen [60]. In contemporary studies, Shinohara’s group used autoantibodies from a patient with age-related cataract to isolate a cDNA encoding a putative lens epithelial cell (LEC) growth factor which was designated LEDGF/p75 [19]. They subsequently showed that the gene encoding the LEDGF/p75 protein (aa 1–530) also encodes a smaller alternative splice variant called LEDGF/p52 (aa 1–333), and sequence analysis revealed identity to the transcription co-activators p75 and p52 [61]. Initial studies from this group provided evidence that LEDGF/p75 is a stress response protein that protects LECs and other ocular cells from environmental stressors by transactivating stress survival genes [18,19,23,24,25,26,27,28,29,30,31,32].

Shortly after these initial studies, the groups of Debyser, Engelman, and Poeschla reported independently that during HIV-1 infection, LEDGF/p75 interacts specifically with HIV-IN to facilitate viral integration into the host DNA [62,63,64,65,66,67,68]. This seminal observation catapulted LEDGF/p75 to the limelight in the field of HIV/AIDS research due to its role as a key cellular co-factor for HIV-1 integration and replication. It also paved the way for a plethora of molecular studies that uncovered not only its mechanistic role in HIV integration but also many aspects of its biological functions in normal and malignant cells as well as its potential as a therapeutic target.

## 3. LEDGF/p75 Structural Domains and Functions

### 3.1. N-Terminal PWWP Domain

The gene encoding LEDGF/p75 has been designated *PSIP1* (PC4 and SFRS1 Interacting Protein 1) [69]. While the LEDGF/p75 and PSIP1 names are commonly used in the cancer, eye disease, and HIV literature, DFS70 is mostly used in the autoimmunity literature. *PSIP1* has been mapped to chromosome 9p22.2 and encodes various splice variants of LEDGF/p75, with p75 and p52 being the best characterized [17,40,61,70,71]. LEDGF/p75 belongs to the hepatoma derived growth factor (HDGF) family of chromatin binding proteins [72]. These proteins share significant sequence homology, particularly in their N-terminal region, which contains a PWWP domain, named after its conserved Pro-Trp-Trp-Pro sequence motif found, that binds to nucleosome structures in double-stranded DNA (ds-DNA) [72,73,74]. Over 20 proteins containing PWWP domains have been identified including, in addition to LEDGF/p75 and p52, HDGF, HDGF-related protein 2 (HRP-2, also known as HDGF2, HDGFL2, and HDGFRP2), DNA methyl transferases (DNMTs), and nuclear SET domain-containing (NSD) proteins [72,73,74,75,76].

**Figure 1:**
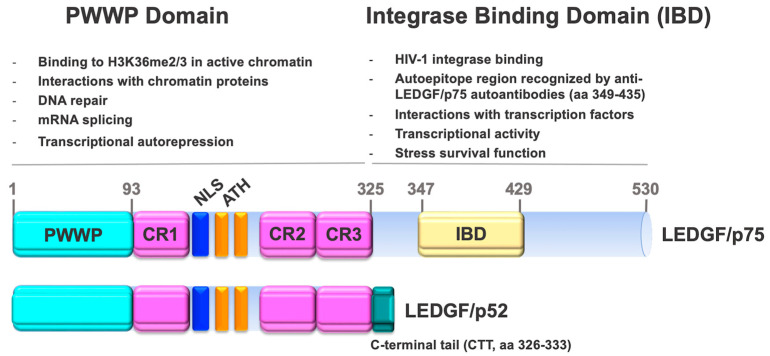
LEDGF domains and functions. The N-terminal region of LEDGF/p75 (aa 1–530) and its short variant p52 (aa 1–325) contains a PWWP domain, three charged regions (CR), nuclear localization signal (NLS), and AT-hooks (ATH). The C-terminal region of LEDGF/p75 (aa 325–530) contains the HIV integrase binding domain (IBD, aa 347–429), which is targeted by autoantibodies in certain individuals. The IBD is absent in LEDGF/p52, which instead has a unique 8-amino acid stretch called the carboxy-terminal tail (CTT, aa 326–333). The functions of both domains are listed.

The PWWP domain of LEDGF/p75 and p52, facilitates their reading of the methylated histone marks H3K36me2 and H3K36me3 in active chromatin, making both variants epigenetic readers (Figure 1) [77,78,79,80,81]. H3K36me2/3 participate in the recruitment of DNMTs, which are required for the maintenance of CpG methylation at intergenic DNA regions and implicated in various human developmental disorders and cancers [82].

LEDGF/p75 and p52 were recently shown to bind to R-loops at RNAPII transcription sites to facilitate transcription, suggesting a role for the PWWP domain in this process [83]. R-loops, comprised of RNA-DNA hybrids, are essential for DNA replication, transcription initiation and termination, and DNA repair [84,85]. However, their unscheduled, unresolved accumulation leads to prolonged RNAPII pausing and transcriptional arrest, leading to DNA damage, genomic instability, and subsequent inflammation [83]. LEDGF/p75 depletion leads to accumulation of R-loops and DNA damage at gene promoters or transcription start sites, increasing the sensitivity of PCa cells to the poly (ADP-ribose) polymerase 1 (PARP1) inhibitor Olaparib and DNA damaging chemotherapeutic drugs such as etoposide [83]. These findings implicated LEDGF/p75 in promoting transcription elongation, genomic stability, and resistance to DNA damaging drugs.

### 3.2. C-Terminal IBD Domain

The C-terminal region of LEDGF/p75 (aa325–530), absent in p52, contains the HIV-IN binding domain (IBD, aa347–429), critical for facilitating HIV integration and oncogenic transcription factors into active chromatin sites (Figure 1). The IBD is a large protein binding hub capable of accommodating interactions between LEDGF/p75 and multiple transcription factors and other chromatin-associated proteins [21,22,50]. Like many other nuclear proteins targeted by human autoantibodies, LEDGF/p75 contains intrinsically disordered structures (IDRs), one between the PWWP and IBD domains and another at the extreme C-terminus downstream the IBD domain [21,55,86]. These IDRs may provide the conformational flexibility necessary for facilitating protein-DNA and protein-protein interactions and could make LEDGF/p75 more susceptible to proteolysis [55]. Indeed, while the PWWP domains of both LEDGF/p75 and p52 are disrupted by caspase-mediated cleavage during apoptosis, the IBD remains relatively intact [38,71]. However, the IDR region immediately following the IBD in the extreme C-terminus is cleaved off during apoptosis by caspases-3 and -7, generating LEDGF/p75 variants that contain an intact IBD with a truncated C-terminus lacking amino acids 486–530 [38]. Unlike full-length LEDGF/p75, overexpression of these truncated variants in cancer cells failed to protect against stress-induced cell death [38].

**Figure 2:**
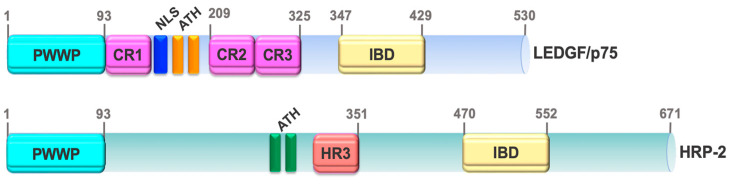
Domain structure of LEDGF/p75 and HRP-2. Both proteins have PWWP and IBD domains as well as AT-hook (ATH) motifs. HRP-2 has a conserved homology region III (HR3) not present in LEDGF/p75. The PWWP and IBD domains have equivalent functions in both proteins.

Although LEDGF/p75 shares amino acid sequence homology with most HDGF proteins, particularly within the PWWP domain [72], only one other member of this family, HRP-2, has both PWWP and IBD domains (Figure 2) [87,88]. The presence of PWWP and IBD domains in HRP-2 allows interactions with LEDGF/p75’s binding partners, including HIV-IN [87,89]. This not only maintains residual HIV-1 integration into active chromatin in LEDGF/p75-depleted cells [90,91,92], but likely complements LEDGF/p75 functions in cancer cells by the ability of HRP-2 to interact with the same oncogenic transcription factors [50,93,94]. Both LEDGF/p75 and HRP-2 promote leukemic survival [94] and cooperate as histone chaperones in the absence of the FACT (facilitates chromatin transcription) complex to relieve nucleosome-induced barrier to RNAPII transcription in differentiated cells [95]. Because of their shared structural and functional features these proteins are considered paralogs [94].

## 4. Stress Survival Functions of LEDGF/p75

LEDGF/p75 is upregulated via DNA methylation upon cellular exposure to agents that induce oxidative or thermal stress, and binds to stress response elements (STRE) and heat shock elements (HSE) in the promoter regions of specific target genes [18,24,26,27,96]. This increases the expression of stress survival proteins such as heat shock protein 27 (HSP27/HSPB1) [28,43,96,97], an oncoprotein that responds to cancer cell insults, including chemotherapeutic stress, by antagonizing caspase activation and apoptosis [98,99]. LEDGF/p75-mediated transactivation of HSP27 is negatively regulated by posttranslational modifications by SUMO enzymes, with SUMOylation at N-terminal residues K75, K250, K254, and C-terminal residue K364, attenuating LEDGF/p75’s transcriptional activity on the *HSP27* promoter [100,101]. However, de-sumoylation of LEDGF/p75 by the sumo-specific protease Senp-1 restores its transcriptional activation of HSP27 [101]. It remains to be determined if this regulatory mechanism also impacts the transcriptional activity of LEDGF/p75 on the promoter regions of other target genes.

Elevated expression of LEDGF/p75 in LECs and cancer cells also upregulates the expression of genes associated with oxidative stress responses including alpha B crystallin (CRYAB/HSPB5), involucrin (IVL), cytoglobin (CYGB), antioxidant protein 2/peroxiredoxin 6 (AOP2/PRDX6), alcohol dehydrogenases (ADH), aldehyde dehydrogenases (ALDH), and the oxidoreductase and glucose-regulated protein GRP58/ERp57/PDIA3 [18,25,26,29,31,34,35]. Thus, LEDGF/p75 transcriptional functions appear to be critical for reducing cellular oxidative damage and evading stress-induced cell death.

RNA sequencing (RNA-seq) studies from our group and others have shown that LEDGF/p75 silencing in cancer cells leads to differential expression of gene pathways associated with oxidative stress response, regulation of apoptosis, cell cycle progression, and DNA repair [49,102]. The ability of LEDGF/p75 to regulate multiple gene pathways that protect cells against environmental stressors that cause oxidative DNA damage and cell death (e.g., radiation and cytotoxic drugs) is highly advantageous to tumors overexpressing this protein, providing a molecular mechanism of therapy resistance.

## 5. The LEDGF/p75 Interactome

**Figure 3:**
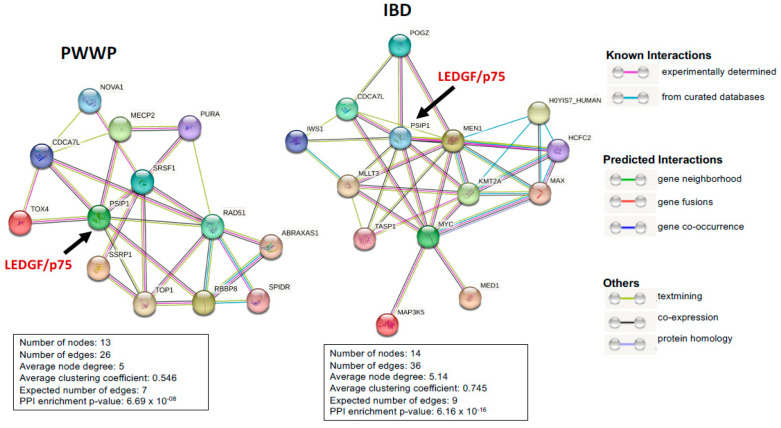
LEDGF/p75 (PSIP1) interaction module for the PWWP and IBD domains generated by STRING analysis (https://string-db.org, accessed on 9 October 2024).

Both the PWWP and IBD domains of LEDGF/p75 participate in protein-protein interactions that regulate essential cellular functions, including mRNA splicing, DNA repair, transcription, and stress survival. STRING analyses of protein-protein interactions involving both domains are shown in Figure 3.

### 5.1. PWWP Domain Interacting Partners

The PWWP domain is an epigenetic reader that also interacts with mRNA splicing factors at transcriptionally active sites to regulate gene splicing [77]. For instance, RNA-seq studies on HEK293T cells with depletion of LEDGF/p75 revealed significant changes in the splicing patterns of over 5000 genes, suggesting a role in alternative splicing regulation [77]. During HIV integration, LEDGF/p75 interacts with multiple mRNA splicing factors to direct integration to transcription units that are highly spliced [103]. These interactions, direct or indirect, likely occur at the PWWP domain given that both LEDGF/p75 and LEDGF/p52 interact with common splicing factors, including SFRS1, SF3B2, hnRNP M, and NOVA1 [103,104,105]. LEDGF/p75’s mRNA splicing regulatory functions allow it to target oncogenic transcription factors to highly spliced genes via IBD-mediated interactions.

Consistent with these studies, mutations in the *MECP2* gene, which encodes the epigenetic reader MeCP2, disrupted the interaction between this protein and LEDGF/p75, resulting in altered mRNA splicing [106]. In a previous study, our group characterized the interaction between MeCP2 and the PWWP domain of LEDGF/p75 in PCa cells, demonstrating their cooperation in enhancing the transactivation of the *HSP27* promoter in cancer cells [97]. Like LEDGF/p75, MeCP2 is an epigenetic reader and emerging oncoprotein in various human cancer types, with primary role in linking DNA methylation to oncogene expression [107]. The PWWP domain of LEDGF/p75 also assists in the recruitment of polycomb group protein BMI1 and co-repressor CTBP1 to mixed leukemia lineage (MLL) transcription complexes in homeobox (*HOX*) gene promoters [108]. Another transcription co-activator, TOX4, also interacts with the PWWP domain in the chromatin of cancer cells [104], although it remains to be established if this interaction modulates oncogenic gene expression.

In addition to modulating alternative splicing, the PWWP domain of LEDGF/p75 interacts with several proteins to enhance the cellular capacity to counter the effects of DNA damaging agents (e.g., radiation and certain anti-cancer drugs) by promoting repair of DNA double strand breaks (DSBs) via the homologous recombination (HR) pathway [47,48]. This function is promoted by the SETD2 methyltransferase, which mediates the conversion of H3K36me2 to H3K36me3, and involves the recruitment through the PWWP domain of the DNA repair proteins CtIP and RAD51 to DSBs in actively active sites [109,110]. LEDGF/p75 and H3K36me3 interact with several DNA damage repair (DDR) proteins in active transcription sites, including PARP1, histone gamma H2A.X, XRCC1, DNA ligase 3, SPT16, Topoisomerases and BAZ1B [80]. LEDGF/p75 depletion in cancer cells decreased recruitment of downstream DDR proteins such as replication protein A 32 kD subunit (RPA32) after exposure to etoposide, and this was considered indicative of inhibition of RPA32-CtIP-BRCA1-mediated homology-directed repair [48]. Interestingly, LEDGF/p75 depletion also led to reduced protein levels of the ubiquitin-conjugating enzyme UBC13 and nuclear proteasome activator PA28γ, suggesting a role in protein degradation events involved in the regulation of DDR signaling molecules [48]. Notably, the FACT complex subunit SSRP1, an interactor of LEDGF/p75’s PWWP domain [111], has also been implicated in promoting DNA repair in human glioblastoma cells [112].

The ability of LEDGF/p75 and its PWWP interacting proteins to resolve R-loops appears to be critical for its DNA repair functions. Recent findings showed that LEDGF/p75, but not LEDGF/p52, is in complex with y-H2AX at DNA damage sites caused by R-loop accumulation at transcription sites [83]. Prostate cells depleted of LEDGF/p75, showed a reduced number of RAD51 foci (marker of the HR pathway), whereas the number of 53BP1 foci and the protein levels of DNA-PK-markers linked to the non-homologous end joining (NHEJ) DNA repair pathway were elevated, consistent with a specific role for LEDGF/p75 in DNA repair via the HR pathway [83]. This study also demonstrated that LEDGF/p75 and PARP1 are interacting partners and bind together to R-loops to promote their resolution and DNA damage repair [83].

### 5.2. IBD Domain Interacting Partners

LEDGF/p75 interacts with several transcription factors, transcription-related kinases, and chromatin remodelers through its C-terminal IBD. These include the mixed leukemia lineage histone lysine methyltransferase protein MLL1 (KMT2A) and its interacting partner Menin, the c-MYC interacting partner JPO2 (R1/CDCA7L/RAM2), the RNAPII associated and RNA processing regulator IWS1, the cell cycle-regulated CDC7-ASK kinase (not to be confused with the ASK1 apoptosis signal kinase), the chromatin remodeling protein POGZ, and the RNAPII transcription mediator complex subunit MED1 [21,22,50]. These proteins interact with the IBD via a disordered IBD-binding motif (IBM) whose phosphorylation regulates their affinity for IBD binding [86].

Contacts between amino acids from the IBD and a downstream C-terminal α_6_-helix have been shown to be critical for LEDGF/p75 dimerization [113,114]. Although this dimerization does not affect LEDGF/p75 capacity to interact with the IBM motifs of its IBD-binding partners, it seems to be important to locally increase the concentration of its interacting transcription factors at active chromatin sites to enhance RNAPII-dependent transcription [114]. This dimerization was stimulated by the presence of DNA, enhanced the binding of LEDGF/p75 to MLL1, and was required for increased colony-forming capacity of MLL1-AF9+ leukemic cells [115]. However, it remains to be explored if this dimerization contributes to the pro-survival functions of LEDGF/p75 in other malignancies, particularly solid tumors.

As mentioned above, HRP-2 also has a C-terminal IBD that enables it to maintain residual HIV-1 integration in the absence of LEDGF/p75 [89,90,91,92]. This compensatory mechanism is facilitated by the interaction between the HRP-2 with HIV-IN and other known LEDGF/p75 IBD interactors such as Menin, MLL1, and IWS1 [50,93,94]. Although there is no direct evidence that HRP-2 and LEDGF/p75 physically interact with each other, our studies revealed their endogenous co-immunoprecipitation and co-localization in docetaxel-resistant PCa cells as part of a large complex localized to active chromatin that also involves several IBD interacting partners [50]. We observed that like LEDGF/p75, HRP-2 contributes to the survival, clonogenicity, and tumorsphere formation capacity of docetaxel-resistant PCa cells [50], consistent with the emerging role of HRP-2 as an oncoprotein that influences epigenetic transcriptional regulation and anti-tumor drug responses in cancer cells [93,94,116,117]. Additional studies are needed to establish if LEDGF/p75 and HRP-2 play complementary and cooperative roles in cancer cells to facilitate the tethering of transcription factors to active chromatin, resolve R-loops, and maintain genomic stability. It also remains to be determined if these functions are cancer cell type-dependent, leading to differential sensitivity to DNA damaging agents.

It has become evident that these interactions not only facilitate the oncoprotein activities of LEDGF/p75 and members of its transcriptional network in cancer cells, but also regulate HIV-1 transcriptional function. For instance, while the LEDGF/p75’s IBD interaction with HIV-IN is critical to promote viral integration at transcriptionally active sites [21,80,103], its binding to the histone chaperone complex IWS1:Spt6 represses HIV-1 transcriptional activity in latently infected cells [118]. This LEDGF/p75:IWS1:Spt6 complex maintains HIV-1 latency by controlling histone H3 occupancy on the long terminal repeat (LPR) sequence associated with repressive nucleosome positioning. It was suggested that this complex could be a cornerstone for the coordination of HIV integration and transcriptional control leading to viral latency in infected cells [118].

## 6. LEDGF/p75 Roles in Cancer Aggressiveness and Chemoresistance

### 6.1. Leukemia

Nucleoporin 98 (NUP98) gene fusions occur with homeobox genes (i.e., *HOXA9*, *HOXD13*) and non-homeobox genes (i.e., *TOP1*, *PSIP1*, *DDX10*) in patients with leukemia [119,120]. Early studies on chromosomal translocations involving the LEDGF/p75-encoding gene *PSIP1* in leukemia were foundational to establish its relevancy in cancer. Several reports showed that fusions between the *NUP98* gene (chromosome 11p15.5) and the *PSIP1* gene (chromosome 9p22.2) give rise to NUP98-LEDGF/p75 fusion proteins with aberrant transcription functions that may influence hematological malignant transformation [121,122,123,124,125,126,127,128]. The *NUP98-PSIP1* gene is formed by the fusion of the IBD-containing C-terminus of LEDGF/p75, where transcription factor binding and pro-survival functions reside, with the N-terminus of NUP98, which localizes to the nucleus and binds to DNA through chromatin binding protein complexes during leukemic transformation [121]. The removal of the PWWP domain of LEDGF/p75 in these fusions likely results in a protein with deregulated transcriptional activity given that it lacks the transcriptional repression functions of this domain [55,129]. The *NUP98-PSIP1* gene fusion is caused by a rare but recurrent chromosomal translocation t(9;11)(p22;p15) reported in adult and pediatric acute myeloid leukemia (AML), chronic myeloid leukemia (CML), and myelodysplastic syndrome [121,122,123,124,125,126,127,128]. Patients carrying this fusion had high mortality, suggesting that the presence of this fusion could be a marker of poor prognosis [123,128].

Although the molecular mechanism underlying the progression and aggressiveness of leukemias with *NUP98-PSIP1* gene fusions is still not clear, it was suggested that they induce a pre-leukemic phase and that additional mutations in other cancer genes are required for progression to AML [126]. In addition, FXFG repeats in the NUP98 N-terminal portion of the fusion gene can act as transactivation domains [121]. Interestingly, a *NUP98-PSIP1* gene fusion was identified in a patient with AML that gave rise to three different fusion transcripts linked to alternative splicing in the *PSIP1* exon 11 [130]. These fusions remained detectable after intensive induction chemotherapy but were undetectable once the patient achieved complete remission following allogeneic stem cell transplantation, suggesting that monitoring *NUP98-PSIP1* fusion transcripts is critical for evaluating residual disease in chemotherapy-treated leukemias with poor prognosis.

The mechanistic role of LEDGF/p75 in promoting leukemia was initially studied in the context of mixed lineage leukemia (MLL). Yokoyama and Cleary [131] showed that LEDGF/p75 interacts with the Menin-MLL1 transcription factor complex and tethers it to active chromatin to promote leukemogenesis. These investigators demonstrated that the interaction between Menin and LEDGF/p75 recruits MLL proteins that are then positioned into RNAPII complexes in active chromatin by the PWWP domain to induce the expression of cancer-related genes (e.g., *HOXA9*). Analysis of the crystal structure of Menin with a MLL1-LEDGF/p75 heterodimer showed that this protein is critical to assemble a Menin-MLL1-LEDGF/p75 ternary complex that regulates gene transcription and promote MLL leukemogenesis [132,133]. LEDGF/p75 knockout was found to inhibit disease progression in MLL murine models, suggesting a critical dependence of MLL leukemogenesis on the transcriptional and pro-survival functions of this protein, implicating it as a potential therapeutic target for leukemias [134].

Van Belle et al. [94] reported that like LEDGF/p75, HRP-2 also interacts with both Menin and MLL1. Mice with a systemic *HRP-2* gene knockout showed no significant differences in white and red blood cell counts compared to wild type cells, although they did show an increase in neutrophils in the peripheral blood [94]. However, lineage depleted bone marrow cells from the knockout mice showed reduced colony formation capacity with an induced myeloid differentiation transcriptomic program, suggesting that HRP-2 is involved in maintaining the stem-like properties of bone marrow cells [94]. Moreover, depletion of HRP-2 in leukemic cells impaired their clonogenic growth, whereas its enforced expression in LEDGF/p75-depleted leukemic cells harboring the MLL1-AF4 fusion rescued clonogenic growth [94]. Consistent with these results, we reported that HRP-2 depletion in docetaxel-resistant PCa cells led to diminished expression of the stem cell marker CD44, suggesting a potential role in cancer stemness [50]. In their study, Van Belle et al. [94] also showed that leukemic cells harboring an HRP-2 knockout can be efficiently transformed by the MLL1-ENL fusion, concluding that while HRP-2 is important for leukemic cell survival, unlike LEDGF/p75, it is dispensable for MLL1-ENL driven leukemogenesis. These studies opened new avenues in the study of leukemogenesis, with targeting LEDGF/p75 and HRP-2 as a potential therapeutic strategy for mixed lineage leukemia.

LEDGF/p75 contributes to chemotherapy resistance in leukemia. Huang et al. [40] showed that LEDGF/p75 transcript was consistently upregulated in blasts from chemoresistant AML patients, and its ectopic overexpression in leukemic cells conferred protection against apoptosis induced by daunorubicin. More recently, Canella et al. [135] reported that LEDGF/p75 depletion in mixed lineage leukemia and other leukemic cell lines increased sensitivity to the chemotherapeutic drug cytarabine. These investigators proposed that the chemoresistance-promoting functions of LEDGF/p75 in leukemia are possibly driven via a mechanism involving regulation by this protein of its IBD-interacting partner MED1 and the bromodomain protein BRD4, resulting in stable and active nuclear super-enhancers, leading to the activation of gene pathways associated with cell cycle checkpoints, cell survival, and stem cell renewal.

While these studies point to a role for LEDGF/p75 in promoting leukemogenesis and leukemic cell chemoresistance, it should be noted that this oncoprotein may also have dual roles in certain leukemias. For instance, a recent study by Demoen et al. [136] showed that knockout of the *PSIP1* gene encoding LEDGF/p75 in spontaneous mouse models of T cell acute lymphoblastic leukemia (T-ALL) accelerated T-ALL initiation in mice, which correlated with altered H3K27me3 signaling. However, depletion of LEDGF/p75 in several T-ALL cell lines led to decrease in cell proliferation associated with induction of apoptosis in certain cell lines. This effect was independent of the presence of MLL1/KMT2A rearrangement, T-ALL subtype, or species-specific differences (mouse vs. human) [136]. Mechanistically, the LEDGF/p75-dependent effect on T-ALL proliferation was linked to downregulation of cytochrome C oxidase assembly factor 20 (COX20), critical for mitochondrial oxidative phosphorylation [136]. The authors concluded that LEDGF/p75 can exert a dual role in the context of T-ALL, either as a tumor suppressor gene during leukemia initiation or as a dependency factor in leukemia maintenance [136].

### 6.2. Prostate Cancer

The identification by our group and others that LEDGF/p75 is a PCa-related tumor associated antigen (TAA) [39,137,138,139] paved the way for further studies on the role of this protein in PCa. Our initial studies revealed that LEDGF/p75 is highly expressed in PCa cell lines compared to primary, non-malignant prostate epithelial cells [39]. Consistent with this, moderate to high expression of this protein was observed in PCa and BPH tissues compared to normal prostate tissues from non-cancer patients [39]. In a subsequent pan-cancer study, we analyzed LEDGF/p75 transcript and protein expression across 21 different cancer types and observed significantly elevated transcript and protein expression of LEDGF/p75 in prostate, thyroid, breast, colon, and uterus, compared to normal control tissues [44]. An important observation was that while the expression of LEDGF/p75 protein, detected by immunohistochemistry (IHC), was low in normal prostate tissues from non-cancer individuals, its expression in “morphologically normal” tissue adjacent to primary prostate tumors was moderate to high [44]. This suggested that a pro-inflammatory prostate tumor microenvironment may lead to the upregulation of this and other oncoproteins not only in the tumor tissues but also in adjacent normal tissues via field cancerization. This is a critical consideration when analyzing LEDGF/75 expression in cancer tissues because bioinformatic analyses of cancer gene datasets comparing *PSIP1* transcript expression in tumor tissues vs. control tissues may not reveal significant differences if the latter were derived from “morphologically normal” areas adjacent to the tumor. Furthermore, as discussed below, LEDGF/p75 expression may increase during tumor progression in response to therapy.

The upregulation of LEDGF/p75 in clinical prostate tumors suggested a role in protecting PCa cells against stressors impacting the tumor microenvironment, including chemotherapeutic drugs. Consistent with this, our group observed that enforced overexpression of LEDGF/p75 in PCa cells protected against docetaxel-induced lysosomal cell death and oxidative-stress induced necrosis, but not against classical apoptosis inducers such as tumor necrosis factor related apoptosis inducing ligand (TRAIL) or staurosporine, suggesting selectivity in its pro-survival activity [46]. LEDGF/p75 knockdown, on the other hand, decreased the clonogenicity of docetaxel-resistant PCa cells in the presence of the clinically relevant taxane drugs docetaxel and cabazitaxel [140]. Enforced expression or knockdown of LEDGF/p75 in PCa cells followed by pathway specific gene profiling also identified a number of differentially expressed antioxidant and stress survival genes [34]. These included *CYGB*, *SOD3*, *TPO*, *GSTZ1*, *PDLIM1*, *PIP3-E*, and *SGK2*.

We also identified HSP27, implicated in taxane resistance [98,99], as a target gene of the LEDGF/p75-MeCP2 interaction in PCa cells [97]. Consistent with this, Barghavan et al. [43] reported that depletion of LEDGF/p75 in PCa cells attenuated HSP27 expression and inhibited their aggressive properties, including proliferation, survival, invasion, and migration. These investigators also showed that LEDGF/p75 silencing in PCa cells results in decreased expression or activation of cellular survival proteins including Bcl-2, Bcl-X_L_, cyclin B1, and the ERK/AKT pathway [43]. Together, these results suggested a transcriptional mechanism by which LEDGF/p75 protects PCa cells by interfering with stress-induced cell death via upregulation of pro-survival and antioxidant genes.

Our recent study showed that LEDGF/p75 and several members of its IBD interactome (Menin, MLL1, JPO2, MED1, POGZ, IWS1, and CDC7-ASK) are upregulated in PCa cells that transitioned to docetaxel resistance [50]. These proteins interact in the nuclei of chemoresistant cells as part of a transcription complex that also includes c-MYC (a JPO2-interacting protein), HRP-2, and the active chromatin marker H3K36me2 [50]. Interestingly, c-MYC, a key driver of cancer stem cells and metabolism, was a top upregulated gene in docetaxel-resistant PCa cells that also displayed increased markers of epithelial-mesenchymal transition (EMT), stemness, and metabolic genes [141]. Depletion of LEDGF/p75, JPO2, Menin, or HRP-2 significantly decreased the survival, clonogenicity, and tumorsphere formation capacity of chemoresistant PCa cells in the presence of docetaxel [50]. However, only depletion of JPO2 and HRP-2 led to a significant decrease in the number of cells expressing the CD44 stem cell marker [50], suggesting that LEDGF/p75 itself may not directly influence stemness in chemoresistant PCa cells.

These studies suggest that LEDGF/p75 and members of its transcriptional network contribute to the aggressive properties of PCa cells, including chemoresistance. This network is likely to drive PCa chemoresistance by activating stress survival and stemness transcriptomic programs. However, it remains to be established which transcription factors drive the expression of LEDGF/p75 and its interactome in chemoresistant cancer cells. Our recent studies implicated GR, a nuclear steroid receptor linked to PCa resistance to both anti-androgen therapy and taxane chemotherapy [142,143,144,145], in the transcriptional activation of LEDGF/p75 as well as the nuclear translocation of its IBD interactor JPO2 in chemoresistant PCa cells [49,50,146]. GR has been found expressed at low levels in primary prostate tumors from PCa patients; however, its expression increases as tumors develop metastasis and resistance to anti-androgen therapy and taxane chemotherapy [8,9,142,143]. This implies that GR is likely to promote increased LEDGF/p75 expression at advanced tumor stages in the context of therapy resistance. GR and LEDGF/p75 also interact in PCa cells and their co-targeting, using selective GR modulators (SGRM) combined with LEDGF/p75 knockdown, decreased the clonogenicity of chemoresistant PCa cells in the presence of docetaxel [49]. Both proteins cooperate to promote docetaxel resistance through the regulation of multiple gene pathways including oxidative stress response, apoptosis regulation, and cell cycle progression [49]. In addition to GR, the transcription factor SP1 has also been established as a driver of LEDGF/p75 expression in various cell types [96,147,148,149], but its role in regulating LEDGF/p75 and other interactome members in specific cancer types remains to be investigated.

### 6.3. Breast Cancer

Our analysis of LEDGF/p75 expression in human cancers revealed significant transcript upregulation in breast tumor tissues [44]. High LEDGF/p75 expression has been documented to play a role in breast cancer (BCa) aggressiveness. Analysis of The Cancer Genome Atlas (TCGA) datasets by Singh et al. [102] revealed LEDGF/p75 upregulation in triple negative breast cancer (TNBC), with high expression significantly correlating with reduced patient survival. In addition, IHC analysis showed increased levels of the LEDGF/p75 protein in metastatic invasive ductal carcinoma. Further, these investigators showed that LEDGF/p75 expression is upregulated in a panel of BCa cell lines, particularly highly tumorigenic and TNBC cell lines [102]. In addition, LEDGF/p75 depletion reduced the aggressive properties of BCa cell lines including increased cell proliferation, clonogenicity, migration and invasion, whereas its ectopic overexpression promoted these properties [102]. Gene profiling analysis and chromatin immunoprecipitation (ChIP)-qPCR revealed that LEDGF/p75 regulates in BCa cells genes associated with cell proliferation and cycle progression (e.g., *Cyclin D2*, *CDK4*, *CDK6*, *CDC25A*, and *P21*) [102]. Intriguingly, LEDGF/p75 depletion in these cells did not downregulate *HSP27* and *VEGF-C*, two known target genes of this protein in other cancer cell types, suggesting that LEDGF/p75 may contribute to the regulation of *HSP27* and other gene pathways in a cancer cell-dependent manner [102]. Alternatively, while LEDGF/p75 overexpression in cancer cells may lead to transactivation of *HSP27* and other genes, its depletion may not dramatically reduce their expression if other transcription factors are also involved.

Consistent with these studies, Daugaard et al. [41], reported increased transcript expression of LEDGF/p75 in primary and metastatic BCa as well as in bladder cancer tissues. These investigators also observed that cell death induced by HSP70 depletion in a panel of cell lines that included BCa cells is associated with LEDGF/p75 downregulation. To assess the role of LEDGF/p75 in cancer chemoresistance, they ectopically overexpressed this protein in MCF7 BCa cells and other cancer cell lines and noticed enhanced lysosomal stability and protection against anti-tumor drugs that induced lysosomal membrane permeabilization (LMP) and DNA damage such as siramesine, BAMLET (a complex of alpha lactalbumin and oleic acid), etoposide and doxorubicin [41,150]. In agreement with our observations in PCa cells [35], LEDGF/p75 overexpression in BCa cells failed to protect against the apoptosis inducer staurosporine [41]. This selectivity for protection against drugs that induce LMP and DNA damage, but not against classical inducers of apoptosis such as staurosporine and TRAIL, could be attributed to the fact that LEDGF/p75 is functionally inactivated by caspase-mediated cleavage during apoptosis, with minimal or no cleavage during lysosomal cell death or other types of non-apoptotic cell death [35,38]. Daugaard et al. [41] also showed that clones overexpressing LEDGF/p75 had increased tumor growth in xenograft models, consistent with its role in promoting cell proliferation. In subsequent studies, they provided evidence that LEDGF/p75 interacts and cooperates with CtIP to promote DNA double strand break repair via HR at chromatin active sites, resulting in increased survival of BCa and other cancer cell types in the presence of DNA damaging therapeutic agents such as ionizing radiation, camptothecin, and mitomycin [47].

Another study reported that cellular stress and LEDGF/p75 overexpression induce *FBXO10*, a ubiquitin ligase and candidate BCa susceptibility gene [151]. In addition, induction of STAT3β, a pro-apoptotic STAT3 splicing variant, in BCa cells repressed the expression of LEDGF/p75, linking this protein to STAT3 signaling in BCa [152].

### 6.4. Ovarian Cancer

Cohen et al. [37] observed that ectopic LEDGF/p75 expression mediated the transcriptional activation of VEGF-C in glioma and non-small cell lung carcinoma cells in vitro, and stimulated VEGF-C expression and augmented angiogenesis and lymphangiogenesis in subcutaneous mouse tumor xenografts. By contrast, LEDGF/p75 knockdown decreased VEGF-C expression. These investigators proposed that LEDGF/p75 transactivates the *VEGF-C* gene by binding to a conserved STRE sequence in its promoter region. Subsequently, the same group demonstrated that the follicle-stimulating hormone (FSH) increased both LEDGF/p75 and VEGF-C expression in ovarian carcinoma cells, and augmented LEDGF/p75 binding to STRE in the *VEGF-C* promoter region [42]. By contrast, LEDGF/p75 depletion decreased hormonally induced expression of VEGF-C. To explore the effect of menopause-induced ovarian failure, these investigators implanted an ovarian cancer cell line expressing the *VEGF-C* promoter in ovariectomized and control mice, observing increased *VEGF-C* promoter activity in the ovariectomized group [42]. Consistent with this observation, the transcript and protein expression levels of both VEGF-C and LEDGF/p75 were higher in the tumors of ovariectomized mice compared to tumors in control mice. Interestingly, the ovarian tumor xenografts in the ovariectomized mice displayed increased lymphangiogenesis and angiogenesis [42]. These results suggested a role for elevated gonadotropin stimulation of LEDGF/p75-mediated VEGF-C expression in augmenting ovarian tumor lymphangiogenesis and angiogenesis in postmenopausal women. Consistent with these studies, another group reported that the lipid growth factor lysophosphatidic acid upregulates VEGF-C expression in PCa cells by activating oxidative stress survival pathways involving LEDGF/p75 and AKT [153].

Intriguingly, recent genomic studies have identified the chromosome locus 9p22.2, where the *PSIP1* gene resides [61], as an ovarian cancer susceptibility locus in BRCA1 and BRCA2 mutation carriers [154,155], consistent with the role of LEDGF/p75 in DNA damage repair. Although our previous pan-cancer expression study did not detect significant LEDGF/p75 overexpression in ovarian cancer [44], it would be of interest to determine if this protein is upregulated in a subset of ovarian tumors contributing to resistance to the DNA damage-inducing chemotherapeutic drugs cisplatin, carboplatin, and Olaparib.

### 6.5. Cervical Cancer

The HPV viral oncogenes *E6* and *E7* are critical for maintaining the malignant phenotype of HPV-positive cervical cancer via their ability to regulate cell death-survival decisions in tumor cells [156]. The role of LEDGF/p75 in cervical cancer was uncovered by Leitz et al. [36], who observed that knockdown or overexpression of *E6/E7* led to LEDGF/p75 downregulation or upregulation, respectively, in cervical cancer cells and human keratinocytes. These investigators also reported that LEDGF/p75 contributes to cervical cancer chemoresistance since its silencing in HPV-positive cervical cancer cell lines reduced colony formation in the presence of the DNA-damaging agents hygromycin B and camptothecin, consistent with the role of this protein in DNA repair [36]. Further, IHC analysis revealed that increased expression of LEDGF/p75 in clinical cervical cancer tissues correlated with HPV-positive lesions [36]. These authors concluded that *E6/E7*-dependent maintenance of intracellular LEDGF/p75 expression is critical for protecting HPV-positive cervical cancer cells against cellular stress, including DNA damage, supporting chemoresistance and tumor cell survival. Mac et al. [110] also demonstrated that SETD2 supports productive HPV replication by promoting DNA repair through the recruitment of LEDGF/p75, CtIP, and Rad51. Another study reported that LEDGF/p75 transcript and protein expression levels are significantly higher in cervical tumors compared to control tissues and correlated with lower patient survival rate [157]. Together, these studies implicated LEDGF/p75 in cervical cancer progression and provided the foundations for the development of novel treatment strategies targeting this oncoprotein to decrease cervical tumor growth and chemoresistance.

### 6.6. Pediatric Brain Tumors

Huang et al. [158] reported that JPO2, a LEDGF/p75-IBD interacting partner, acts cooperatively with the c-MYC oncoprotein to potentiate its transforming activity in mammalian cells, including medulloblastoma cells. They also showed that overexpression of JPO2 in medulloblastoma cells enhanced colony formation whereas its knockdown reduced it [158]. IHC analysis in medulloblastoma tumor tissue microarrays showed tumor-specific expression of JPO2, with modest association with metastatic tumors. A subsequent study by the same group reported that JPO2 and LEDGF/p75 physically interact in medulloblastoma cells and are concordantly upregulated in both human and murine medulloblastoma tissues as well as cell lines [45]. Both proteins also promoted medulloblastoma cell migration and invasion via activation of the PI3K/AKT signaling pathway, critical for medulloblastoma metastasis [45]. Although the contribution of JPO2 and LEDGF/p75 to medulloblastoma chemoresistance was not explored in these studies, both proteins were implicated as novel modulators of the c-MYC/PI3K/AKT signaling axis involved in medulloblastoma aggressiveness and metastasis. These studies also highlighted the potential of targeting the JPO2-LEDGF/p75 interaction for reducing the aggressiveness of medulloblastoma tumors.

In a separate study, Yu et al. [79], investigated the role of H3K36me2, LEDGF/p75, and HRP-2 in diffuse pontine glioma (DIPG), a very aggressive and deadly pediatric brain tumor. Co-depletion of LEDGF/p75 and HRP-2 decreased the proliferation of DIPG cells carrying an H3K27 driver mutation that results in global loss of H3K27me3 but increase in H3K36me2 [79]. Interestingly, this co-depletion alone also led to partial reduction of H3K27M-DIPG tumors and increased mice survival in xenograft models, suggesting that co-expression of LEDGF/p75 and HRP-2 contributes to the growth of DIPG tumors carrying this mutation. The cancer-promoting activity of LEDGF/p75 and HRP-2 is likely mediated by enhanced transcription driven by their cooperative functions and enrichment at H3K36me2 sites in active chromatin.

### 6.7. Esophageal Squamous Cell Carcinoma

Guo et al. [159] identified an STRE element in a single nucleotide polymorphism (rs2395655, −809G/A) in the promoter region of *P21/WAFI/CIP1*, a modulator of the DNA damage response. Ectopic expression of LEDGF/p75 in esophageal squamous cell carcinoma (ESSC) cells stimulated transcriptional activity of this promoter and increased levels of the *P21* transcript. However, its knockdown in ESSC cells harboring the rs2395655 GG genotype, but not the AA genotype, resulted in decreased levels of this transcript. Interestingly, this rs2395655 variant correlated with elevated P21 protein expression in clinical ESSC tissues, which was associated with positive postoperative prognosis [159]. These results were consistent with the previous observation that *P21* gene expression is downregulated in BCa cells depleted of LEDGF/p75 [102]. While this study demonstrated that LEDGF/p75 regulates *P21* transcript expression in ESSC cells by binding to an STRE in the rs2395655 G allele-containing *P21* promoter, a key limitation was the lack of an IHC analysis validating and correlating the expression of these two proteins in clinical ESSC tissues. Further studies would be needed to establish a role for LEDGF/p75 in ESSC progression and therapy resistance.

### 6.8. Renal Carcinoma and Other Cancers

Kanu et al. [160] provided evidence that loss-of-function of SETD2 in renal carcinoma (RCC) is associated with reduced chromatin binding of H3K36me3 and LEDGF/p75, leading to increased DNA damage due to the failure of LEDGF/p75 and RAD51 to load into DSB sites. A SETD2-dependent role of H3K36me3 in DNA repair was identified in these studies, reflecting the requirement of this trimethylated histone for the recruitment of the LEDGF/p75-CtIP complex to DSBs as a mechanism to promote HR. Further, IHC studies showed correlation between SETD2 and H3K36me3 expression in RCC tissues, with tumors harboring SETD2 loss showing reduced H3K36me3 expression and displaying elevated DNA damage [160]. However, the expression of LEDGF/p75 in RCC was not assessed in that study. These results implicated SETD2, H3K36me3, and LEDGF/p75 in maintaining genome integrity in RCC in part by contributing to DNA repair via HR.

A recent pan-cancer bioinformatics study by Zhang et al. [51] revealed that LEDGF/p75 transcript and protein expression are differentially regulated in multiple primary cancers, with increased expression in some tumors (e.g., head and neck squamous cell carcinoma, [HNSC], and thymoma) and downregulation in others (e.g., BCa and PCa), and some cancers showing opposite expression of transcript and protein levels in the same tumor type. This study also showed high expression of LEDGF/p75 in patients with clear cell renal carcinoma (ccRCC) associated with poor overall survival [51]. High LEDGF/p75 expression was also observed in ccRCC cell lines, and knockdown of this protein in these cells reduced their proliferation and migration, and transcriptionally altered several gene pathways including the Wnt and p53 signaling pathways [51]. A limitation of this study, however, was the lack of experimental verification by IHC using tumor samples from different stages and treatments.

## 7. LEDGF/p75 Potential as Oncotherapeutic Target

As discussed above, multiple studies using pre-clinical cancer cellular models have shown that genetic silencing of LEDGF/p75 sensitizes cells to various classes of chemotherapeutic drugs including DNA damaging agents, PARP inhibitors, antimetabolites, and microtubule-stabilizing taxanes [36,40,41,46,47,48,49,83,135]. In addition, LEDGF/p75 silencing attenuates tumor cell aggressive properties by reducing cancer cell proliferation and survival, clonogenicity, tumorsphere formation capacity, migration, invasion, and tumor growth. Given LEDGF/p75’s critical functions and protein interactions in cancer cells that promote tumor aggressiveness, there is a compelling argument for targeting this oncoprotein for cancer treatment, particularly for attenuating chemotherapy resistance.

Currently, there are no specific LEDGF/p75 small molecule inhibitors available that could be evaluated for their potential in cancer treatment. However, recent intense efforts in identifying or designing inhibitors that interfere with the interaction between HIV-IN and the LEDGF/p75 IBD [161,162,163,164] promises to yield novel LEDGF/p75-targeting compounds that could potentially be repurposed for cancer treatment. Early work in this area from the groups of Neamati and Debyser identified N-acylhydrazones and hydrazines as potential compounds using LEDGF/p75 IBD-pharmacophore models [164]. Some of these compounds inhibited the interaction between the LEDGF/p75 IBD and the HIV-IN at low μM concentrations and showed significant cytotoxicity in human colon cancer cells [164], highlighting their potential as anti-cancer agents.

The development of LEDGINs (inhibitors of LEDGF/p75—IN interaction) began with structure-based drug design targeting this interaction, resulting in the identification of 2-(quinolin-3-yl) acetic acid compounds that inhibited HIV replication [165]. Several derivatives were designed based on this structure such as the LEDGINS-1,2,3, CX series compounds, and BI-series compounds that displayed varying IC50 values in their inhibition of the LEDGF/p75—IN interaction in vitro or viral replication [165,166,167]. As interest in LEDGINs grew, studies were expanded to include benzene scaffolds or the N-acylhydrazone and hydrazine scaffolds mentioned above [164,168].

George et al. [169] also developed derivatives from halogen-containing quinoline molecule to synthesize a series of 1,2,3,4-tetrahydroisoquinolines, some of which showed effective inhibition of the interaction between the LEDGF/p75 IBD and HIV-IN in vitro at relatively low μM concentrations. The lead compound from these studies, **6d**, inhibited the interaction efficiently by binding to the catalytic core domain of HIV-IN, which is critical for binding to the IBD. In another study, cyclic peptide inhibitors that bind to the LEDGF/p75 IBD were identified by phage display [170] and showed the ability to inhibit the LEDGF/p75 IBD-IN interaction in vitro. However, these cyclic peptides failed to inhibit LEDGF/p75 functions, lessening their potential use as anti-cancer agents.

Various LEDGINs with potential for inhibiting HIV integration and replication advanced to clinical trials (e.g., STP0404 and *BI224436)* [166,171], although the trials were halted for *BI224436.* Gilead acquired the license for *tert*-butoxy-(4-phenyl-quinolin-3-yl)-acetic acids (tBPQA), which are LEDGINs analogs with potent in vitro antiretroviral activity [172]. These compounds were found to interact with HIV-IN at the IBD binding pocket and showed potent antiviral effects mediated by their competition with LEDGF/p75, which would disrupt chromatin tethering of IN, and induction of conformational change in the IN dimer, which inhibits proper assembly of host DNA-IN complex [172].

A very recent study by Vantieghem et al. [173] conducted a rational-based design study to identify compounds that can bind to the PWWP domain of LEDGF/p75 and inhibit its interaction with the epigenetic marker H3K36me2/3. Using an X-ray crystallography fragment-based screening approach for the HRP-2 PWWP domain (which shares sequence homology with the LEDGF/p75 PWWP domain), they were able to identify 68 chemically diverse hits that were capable of binding to the PWWP H3K36me2/3 pocket at μM concentrations. Further development of these compounds could yield compounds with high affinity and specificity that may prevent the binding of LEDGF/p75 and its paralog HRP-2 to active chromatin in the context of treatment of HIV/AIDS and cancer.

**Figure 4:**
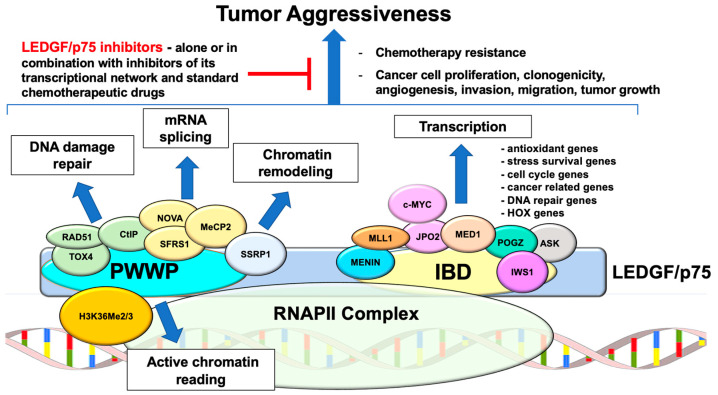
Multiple functions of LEDGF/p75 in cancer and potential as oncotherapeutic target to attenuate tumor aggressiveness. Interactions of LEDGF/p75 with multiple proteins at its PWWP domain are critical for DNA damage repair through homologous recombination, regulating mRNA splicing, chromatin remodeling, and reading of active chromatin. Interactions at its IBD domain with oncogenic transcription factors are critical for the upregulation of gene pathways associated with cancer progression and aggressiveness. Targeting LEDGF/p75 with specific inhibitors, alone or in combination with inhibitors of its transcriptional network and standard chemotherapeutic drugs could be a promising strategy to attenuate tumor aggressive properties, particularly resistance to chemotherapy.

While none of these studies have yielded LEDGINs with specific binding to LEDGF/p75 and ability to inhibit the biological functions of this protein, it is imperative to continue designing and characterizing next generation LEDGINs and other specific-LEDGF/p75 PWWP or IBD inhibitors. These inhibitors should be evaluated for their competition with PWWP interacting partners (e.g., H3K36me2/3, splicing factors, DNA repair proteins) or IBD partners (e.g., Menin, MLL1, JPO2) and their anti-cancer activities (e.g., inhibition of cell proliferation, survival, clonogenicity, migration, invasion, tumor growth, DNA repair, and resistance to therapy) in pre-clinical models of human cancers. The goal will be to attenuate LEDGF/p75-mediated tumor aggressiveness by using specific small molecule inhibitors (SMIs) of this protein, either alone or in combination with SMIs targeting other members of its transcriptional network (e.g., Menin, MLL1/KMT2A, c-MYC, HRP-2) and standard chemotherapeutic drugs or radiation to circumvent therapy resistance (Figure 4).

## 8. Conclusions and Future Directions

The discovery of LEDGF/p75 twenty-five years ago catapulted this protein into the limelight of eye research, HIV/AIDS, and autoimmunity. However, its roles in cancer and therapy resistance have slowly emerged during the past decade. Compelling evidence indicates that LEDGF/p75 is overexpressed in multiple human cancers and promotes the aggressive properties of cancer cells, including chemotherapy resistance. It remains to be explored, however, if LEDGF/p75 also promotes cancer cell resistance to different radiation therapy modalities and immunotherapies. Further, there is still a need for additional in-depth studies focused on understanding the mechanisms by which this protein exerts its oncogenic functions, including knowledge of its interactions with other oncoproteins and transcription factors, as well as its upstream regulators and downstream targets, in various types of cancer cell models.

Future studies should also examine the expression of LEDGF/p75 in relationship to tumor stage and grade, tumor microenvironment, tumor mutational status, and therapeutic modalities, in large cohorts of racially/ethnically diverse cancer patients with annotated genomics, transcriptomics, and clinicopathological data. This will provide critical information on whether the tumor expression and oncogenic functions of LEDGF/p75 are influenced by cancer type, tumor stage and grade, specific therapies, or even the patient’s race or ethnicity.

Finally, the future availability of novel and specific SMIs of LEDGF/p75 could be potentially beneficial for the treatment of tumors with high expression of this protein, either when used alone or in combination with other SMIs or standard therapies. Pre-clinical studies using patient-derived xenografts and organoids would be essential to provide critical insights beyond those obtained using cancer cell line models on the therapeutic effectiveness of these inhibitors prior to the initiation of clinical trials. Given that LEDGF/p75 knockout in mice has been associated with defects in hematopoiesis, suppression of T-ALL initiation, and developmental craniofacial and skeletal abnormalities [69,134,136], and that this protein plays an important role in protecting ocular cells from environmental stressors [23,24,25,26,27,28,29,30,31,32,33], it would be crucial to ascertain in pre-clinical animal models of cancer if its pharmacological inhibition causes serious side effects. In light of the multiple functions of LEDGF/p75 in health and disease, priority should be given to the development of novel strategies to deliver SMIs targeting this protein specifically to tumor cells without systemically affecting normal organs and tissues. This will be critical to circumvent any potential adverse effects of these inhibitors.

## Data Availability

Not applicable.
